# Effects of improved water, sanitation, handwashing, and nutrition on early childhood IgG immune repertoire development against *Shigella* and enteroinvasive *Escherichia coli* (EIEC)

**DOI:** 10.1128/msphere.00339-26

**Published:** 2026-07-15

**Authors:** Nikolina Walas, Everlyn Kamau, Minlu Zhang, Kathy Kamath, Jack Reifert, John Shon, Shahjahan Ali, Md. Ziaur Rahman, Abul K. Shoab, Syeda L. Famida, Salma Akther, Md. Saheen Hossen, Palash Mutsuddi, Mahbubur Rahman, Jessica A. Grembi, Andrew N. Mertens, Taufiqur R. Bhuiyan, Firdausi Qadri, Edward T. Ryan, Richelle C. Charles, Daniel Leung, Stephen Luby, Audrie Lin, Benjamin F. Arnold

**Affiliations:** 1 School of Public Health, University of California 40289, Berkeley, California, USA; 2 Francis I. Proctor Foundation, University of California, San Francisco, California, USA; 3 Serimmune Inc., Goleta, California, USA; 4 International Centre for Diarrhoeal Disease Research, Dhaka, Bangladesh; 5 Department of Epidemiology, Colorado School of Public Health 144805, Aurora, Colorado, USA; 6 Global Health and Migration Unit, Department of Women’s and Children’s Health, Uppsala University 8097, Uppsala, Sweden; 7 Department of Veterinary and Biomedical Sciences, The Pennsylvania State University 8082, University Park, Pennsylvania, USA; 8 One Health Microbiome Center, Huck Life Sciences Institute, The Pennsylvania State University 8082, University Park, Pennsylvania, USA; 9 Division of Epidemiology, University of California Berkeley 1438, Berkeley, California, USA; 10 Massachusetts General Hospital Harvard Medical School, Harvard T. H. Chan School of Public Health, Boston, Massachusetts, USA; 11 Division of Infectious Diseases, Department of Internal Medicine, University of Utah 7060, Salt Lake City, Utah, USA; 12 Division of Infectious Diseases and Geographic Medicine, Stanford University 6429, Stanford, California, USA; 13 Department of Microbiology and Environmental Toxicology, University of California 542823 , Santa Cruz, California, USA; 14 Department of Ophthalmology, University of California 8785, San Francisco, California, USA; 15 Institute for Global Health Sciences, University of California, San Francisco, California, USA; University of Maryland School of Medicine, Baltimore, Maryland, USA

**Keywords:** immune repertoire, shigella, WASH, serology

## Abstract

We studied the effects of a combined water, sanitation, handwashing, and nutritional supplementation (WSH + N) intervention on *Shigella*/EIEC IgG immune development among Bangladeshi children enrolled in a cluster-randomized trial. We used a bacterial display assay to measure IgG-specific binding to *Shigella*/EIEC Ipa proteins (IpaA, IpaB, IpaC, IpaD, and IpaH) from a substudy of 120 children (60 intervention, 60 control) at median ages 3, 14, and 28 months. We found that the WSH + N intervention did not impact IgG seroprevalence (56% vs 53%, *P* = 0.6) or IgG epitope repertoire development (*P* = 0.15–0.97), but there were distinct age-based patterns of IgG linear epitopes and motifs charting the development of the immune repertoire. Samples from before age 6 months, reflecting predominantly maternal IgG, had higher epitope breadth and magnitude compared with samples from older ages. A modeling analysis of maternal-child IgG dynamics suggests that peak susceptibility occurs between 6 and 9 months of age. With this methodology, we found that intensive, household-based WASH interventions were insufficient to impact early childhood immune development to *Shigella*/EIEC. Our results motivate complementary interventions, such as vaccines, to prevent *Shigella*/EIEC infection in the first 2 years of life.

**IMPORTANCE:**

*Shigella*/EIEC is a leading cause of global diarrheal deaths, especially in low-resource settings. Current interventions against childhood *Shigella*/EIEC infection focus on reducing exposure, such as through improved water, sanitation, handwashing, and nutritional supplementation (WSH + N). It is unclear how these interventions impact childhood immune development against pathogens. In the present study, we characterize the null impact of a WSH + N intervention on IgG immune responses to *Shigella*/EIEC in a birth cohort and demonstrate the development of immune responses over the first 2 years of life. We found that maternal IgG wanes between 6 and 9 months, and that peak susceptibility to infection is during this time. From our findings, we provide a modeling framework that can be used to inform vaccination schedules in early childhood after 6 months.

**CLINICAL TRIALS:**

This study is registered with ClinicalTrials.gov as NCT01590095.

## INTRODUCTION

*Shigella* and enteroinvasive *Escherichia coli* (EIEC) are closely related bacterial pathogens, estimated to cause 111 million cases and 63,000 deaths annually in children under the age of five (1–3). *Shigella*/EIEC are symptomatically and biochemically difficult to distinguish and are often grouped together when evaluating diarrheal causes (4, 5). Antibiotics are the only treatment option due to the lack of an available vaccine. Antibiotic use creates selective pressure for antimicrobial resistance, which is largely driven through horizontal gene transfer in *Shigella*/EIEC species (6, 7). In Bangladesh, the prevalence of resistance to first-choice antibiotics is estimated to be as high as 62%, with an overall prevalence of multidrug-resistant *Shigella* at 33% (6, 8–10). The burden associated with *Shigella*/EIEC and development of antimicrobial resistance motivates preventive interventions to reduce infections in high-transmission settings.

*Shigella*/EIEC is primarily transmitted fecal-orally, and due to its low infectious dose (10–100 bacteria), outbreaks can spread quickly from person to person or through contaminated water in settings with poor sanitation (11, 12). Household-level behavioral interventions, such as handwashing, clean drinking water, and human waste sanitation, have been found to reduce diarrheal incidence (13, 14), although these results have not been consistent across populations in low- and middle-income settings (15, 16). In the WASH Benefits Bangladesh trial, WSH + N interventions did not significantly reduce *Shigella*/EIEC infection at 14 months measured by PCR (17). This null finding may be due to seasonal variability in enteric pathogen prevalence and delayed sample collection between comparison groups due to political unrest, although similar non-significant results were observed in other study populations (18, 19).

Single measures of pathogen presence in stool likely underestimate the cumulative incidence of actual infections. Immunoglobulin G (IgG) antibodies to *Shigella*/EIEC measured in blood are long-lived and provide a cumulative measure of previous exposure that could complement PCR-based measures from stool (20, 21). While conventional methods such as ELISA or multiplex bead assays are effective at detecting IgG antibodies against a set of known antigens, bacterial display assays provide a new opportunity to measure the full breadth and diversity of epitopes that make up the IgG antibody population. Serum epitope repertoire analysis (SERA) leverages randomized peptide display to characterize immunogenic peptide motifs and achieve refined epitope resolution (22, 23). This approach offers insight into patterns of age-dependent immune maturation that may not be captured by conventional serologic assays.

Here, we aimed to assess the impact of the combined nutritional and water, sanitation, and hygiene (WSH + N) intervention from the WASH Benefits Bangladesh trial on *Shigella*/EIEC IgG using a randomized bacterial display assay and the serum epitope repertoire analysis (SERA) platform (24). We hypothesized that improved water quality, handwashing, and sanitation interventions would reduce exposure to *Shigella*/EIEC in early childhood and therefore result in lower IgG immune responses. We characterize the age-specific repertoire of linear epitopes and motifs of IgG antibodies specific for *Shigella* Ipa proteins (IpaA, IpaB, IpaC, IpaD, and IpaH) in children sampled longitudinally at 3, 14, and 28 months. We model the waning of maternally derived antibodies and the subsequent rise in seroprevalence due to natural exposure. Finally, we estimate the window of susceptibility to infection for the study population. Our results provide new estimates of the impact of WASH interventions on *Shigella*/EIEC immune development and could help inform future vaccine timing.

## MATERIALS AND METHODS

### Study design and sample selection

The WASH Benefits Bangladesh trial was a cluster-randomized trial to investigate the independent and combined effects of improved child nutrition and household WASH on children’s linear growth and diarrhea. Details for study design and enrollment are described elsewhere (25). In brief, geographically matched clusters were randomized to one of six intervention arms (improved water; improved sanitation; improved handwashing; improved nutrition; improved water, sanitation, and handwashing; or improved water, sanitation, handwashing, and nutrition) or the control arm. Within the larger trial, a substudy focused on environmental enteric dysfunction enrolled 217 clusters from four arms (control; improved water, sanitation, and handwashing; improved water, sanitation, handwashing, and nutrition; and improved nutrition) of the trial and collected venous blood samples from the birth cohort via household visits by field staff at three follow-up visits (median child ages 3 months, 14 months, and 28 months), and plasma samples were stored according to previously published methods (26).

We selected a sample of 120 children for the immune repertoire substudy (Table S1). We restricted our analysis to children that were enrolled in the control group or the combined nutritional and water, sanitation, and hygiene (WSH + N) intervention group (*n* = 900), and had a blood sample collected at all three visits. We excluded children that had their first measurement (ages 1–6 months) between August and November of 2013 to improve comparability between arms in visit timing with respect to calendar date and season, as measurement in control villages was paused during late 2013 due to political instability in Bangladesh. We also restricted the sample to children who were aged between 28 and 200 days at visit 1 and 365 and 548 days at visit 2 to improve balance between the two groups with respect to age and measurement dates. Among 147 children who met inclusion and exclusion criteria, we selected a random sample of 120 children (60 children per group, 360 total samples) for the analysis. The overall sample size was determined primarily based on logistical and cost feasibility for the immune repertoire assay testing. We determined that a sample size of 60 children per group would have 80% power to detect an absolute reduction of 25% seroprevalence with a two-sided alpha of 0.05 and an assumed 50% seroprevalence, using a standard equation for comparison of two proportions.

### Serum epitope repertoire analysis (SERA) platform

The peptide display library was prepared and used for selection according to detailed methods previously published (22, 27). Briefly, the *E. coli* peptide display library was induced for peptide expression, resuspended in a 15% glycerol/PBS solution (PBSG), and aliquoted into deep-well 96-well plates at 10-fold the library diversity in each well (8 × 10^10^ cells per well). Plates were frozen and stored at –80°C for future use. Selection was performed per published protocol using a 1:25 dilution of each sample, capture of antibody-bound bacteria using protein A/G Sera-Mag SpeedBeads (GE Life Sciences, 17152104010350) (6.25% the beads’ stock concentration), and growth of the selected bacteria overnight with shaking (300 rpm) at 37°C.

Plasmids were isolated from the selected bacteria, and PCR was used to amplify the region encoding the random peptides expressed on the cell surface during selection and to incorporate adaptors and indices necessary for pooling samples for NGS sequencing. Details have been previously published (22, 27). Purified amplicons with indices and adaptors were pooled, prepared for sequencing, and sequenced according to the specifications of the Illumina NextSeq 500 using a High Output v2, 75-cycle kit (Illumina, FC-404-2005).

### Bioinformatics

Once samples were processed through the SERA assay and sequenced, they were scored against all previously developed and validated epitope motifs reflective of antibody responses to different exposures. Previously published works have described in detail methods for motif discovery using an algorithm (IMUNE) to create and validate exposure panels (22, 27, 28). Briefly, groups of epitope motifs (panels) that demonstrate high sensitivity, specificity, and breadth of coverage in a discovery disease and control set of samples are selected and compiled together. A control group is used to generate z-scores for every motif and every sample screened through the assay. Individual motif z-scores are summed for each unique epitope to generate a composite score for the exposure for which that motif panel represents. The resulting composite score (SERA score) is used to determine a cutoff for positivity based on predicate-tested disease and controls. A new set of validation samples is then tested and scored to determine performance metrics (sensitivity and specificity) of the motif panel.

A preliminary panel of epitope motifs was discovered for exposure to *Shigella* using the same methods described above with the exception that predicate-tested controls were not utilized. Instead, databased sample data originating from the U.S.A. and provided as part of a cohort of Blood Centers of America (BCA) healthy controls were used in comparison to samples provided for different infectious diseases prevalent outside of the U.S.A. A selection of the samples for those born outside of the U.S.A. (ex-U.S.) were compared to BCA samples to discover motifs that aligned to known *Shigella* antigens (primarily invasion plasmid antigens—IpaB, IpaC, and IpaD) and developed into a panel of epitope motifs as described previously (22). Because no predicate-tested validation samples were available, additional ex-U.S. samples were used to estimate a composite score cutoff of 50 for the panel (exposure panel is preliminary). A plot comparing relative composite scores of ex-U.S. samples to BCA controls is provided with seroprevalence estimates in Fig. S1, and the raw scores of the samples are provided in Data S1. All samples from this study were scored against this preliminary motif panel. Heatmaps of z-scores for each individual motif against every sample are provided in Data S2 to compare relative reactivities across sample sets for each antibody reactive epitope.

To identify linear epitopes, we investigated the Ipa proteome using protein-based immunome-wide association studies (PIWAS) (29). For each sample, approximately 750,000 to 3.6 million unique 12-mer sequences were obtained from the SERA assay, and the sequences were decomposed into constituent 5- and 6-mers. An enrichment score for each 5- or 6-mer was calculated by dividing the number of unique 12-mers containing the kmer by the number of expected 12-mers for the sample, based on the amino acid proportions in the sample. For each sample of the study, 5- and 6-mers were tiled across the protein sequences of the *Shigella* Ipa proteins (A, B, C, D, and H3) (Table S2). These Ipa proteins are highly conserved among *Shigella* species and EIEC and are recognized as key targets in current *Shigella* vaccine development. We used the *S. sonnei* genomic sequences as the representative reference for the tiling process. However, because the Ipa protein sequences cannot be reliably distinguished among different *Shigella* species and EIEC, all assay results were interpreted at the broader *Shigella*/*EIEC* pathovar level (30–32). A tiling enrichment score, sometimes referred to as IWAS score, was calculated at each amino acid position of the protein based on the corresponding 5- or 6-mer enrichment scores, with a smoothing window of five 5-mers and five 6-mers. We then log-transformed these enrichment scores to *z*-scores and normalized them to BCA health population controls as described in (28). The normalized z-scores are hereafter referred to as IgG enrichment values.

### Defining epitopes, seroreactive regions, and summary measures

**Fig 1:**
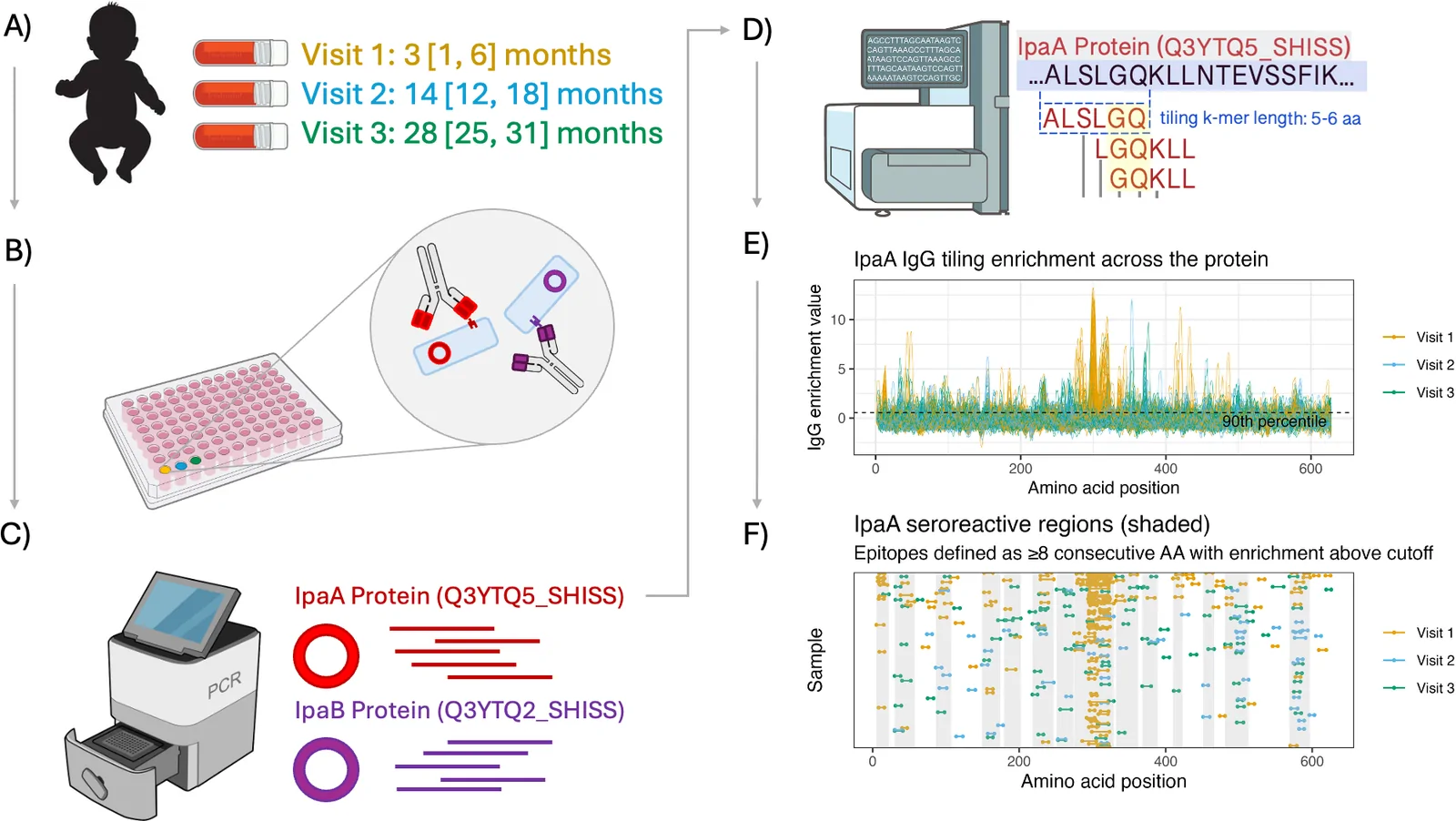
Overview of linear IgG epitope identification. (**A**) Three hundred and sixty serum samples originating from a sample of 120 children were analyzed using a randomized bacterial display assay. (**B and C**) Plasmids expressing protein sequences that bound serum antibodies were purified and sequenced using PCR. (**D**) An IgG tiling enrichment score was calculated for each amino acid position of corresponding *Shigella*/EIEC Ipa proteins (IpaA, IpaB, IpaC, IpaD, and IpaH) using 5-mer and 6-mer log-enrichment with a window size of 5 for smoothing and normalized to healthy controls. (**E**) Epitope hits were defined as eight or more consecutive amino acids with an IWAS score above the 90th percentile for that protein across all 360 serum samples. (**F**) Seroreactive regions were defined as amino acid regions with an overlap of five or more epitope hits from unique samples (1.5% of samples).

We defined epitope hits as eight or more consecutive amino acids with an enrichment tiling score above the 90th percentile of the enrichment score for that protein. Areas of high reactivity per protein, referred to as seroreactive regions, were defined as amino acid regions that contained epitope hits in at least five unique samples. A sample was defined as being seropositive to a protein if one or more epitope hits were identified. Seropositivity to the pathogen was defined as having at least two epitope hits to any of the Ipa proteins included in the panel. An overview of the method is highlighted in Fig. 1. Sensitivity analyses suggest that similar age-dependent patterns were slightly elevated or blunted depending on the chosen threshold for defining seroreactive regions and pathogen-level prevalence. We chose the current threshold as it was an intermediate for our reported estimate and clearly maintained the observed age-dependent pattern in this population (Fig. S2).

To verify our method, we cross-checked our seropositivity assignment with PCR measurements taken at visit 2 (14 months) for this sample set. We defined seropositivity as being seropositive at visit 2 (14 months) or visit 3 (28 months) to allow for seroconversion time. We also compared the preliminary motifs identified using the ex-U.S. data set and applied to the WASH data set with those from known *Shigella* cases and healthy controls enrolled in Dhaka, Bangladesh, at the International Centre for Diarrhoeal Disease Research (icddr,b). Detailed information regarding the case and healthy control data can be found in reference 21. Patients presenting with moderate to severe diarrhea and positive stool culture or PCR for *Shigella*/EIEC were enrolled, and the IMUNE computational algorithm was used to identify *Shigella*-specific motifs, and sensitivity compared to control patients was calculated at days 2, 7, and 30 post-infection. Summary data for this analysis can be found in Data S3.

Quantitative summary measures were defined as the count of epitope hits in seroreactive regions and the mean of enrichment values in seroreactive regions over the entire protein, akin to the outlier sum score proposed by Tibshirani and Hastie (33) for analyses of genomic expression data. To account for proteins with varying amino acid length, the pathogen-level summary of the mean enrichment in seroreactive regions was calculated by taking the mean across the amino acids in seroreactive regions for all proteins in the panel. Total epitope hits in a sample were calculated by summing across all Ipa proteins in the panel.

### Statistical analysis

Participants were analyzed according to their original randomized group (intent to treat). Primary between-group comparisons combined results from visits 2 and 3 at median ages of 14 and 28 months, excluding earlier samples to avoid potential contributions of maternal IgG. We used linear-binomial regression models to estimate the difference in IgG seroprevalence between treatment groups, accounting for repeated outcome measurements using robust standard errors at the cluster level. The same approach was used to estimate the difference in seroconversion per child-year, but we assumed that all children were at risk of seroconversion at visit 2 and that only those who were seronegative at visit 2 were at risk for seroconversion between visits 2 and 3. We used linear regression models to estimate the difference of quantitative summary measures—mean IgG enrichment, maximum IgG enrichment, and the SERA score between groups—while accounting for repeated measures using robust standard errors. Differences in epitope hits between the groups were estimated using a permutation test based on the Mann-Whitney U test statistic, with treatment permuted at the cluster level (10,006 permutations).

We used a modified catalytic model framework to account for passive maternal IgG transfer and IgG developed through natural exposure and estimate age-based *Shigella*/EIEC seroprevalence, as described in depth elsewhere (34). Briefly, the model assumes a constant force of exposure over time and age, defined as the rate at which susceptible individuals become infected (parameter λ), and a constant rate of decay of maternal antibodies (parameter γ) using a closed system of differential equations outlined below:


$$\frac{dM}{da}=-\gamma M(a)$$

$$\frac{dS}{da}=\gamma M(a)-\lambda S(a)$$

$$\frac{dX}{da}=\lambda S(a)$$

The system solution has the following solutions:


$$\begin{array}{rl} M(a) & =e^{-\gamma a}\\ S(a) & =\frac{\gamma }{\left(\gamma -\lambda \right)}(e^{-\gamma a}-e^{-\lambda a})\\ X(a) & =\frac{\gamma (1-e^{-\lambda a})-\lambda (1-e^{-\gamma a})}{\gamma -\lambda } \end{array}$$

where *M*(*a*) is the proportion of children of age *a* losing maternal immunity at a constant rate γ, *S*(*a*) is the proportion of children susceptible among those born with maternal immunity, and *X*(*a*) describes the proportion of children seropositive from natural exposure at a constant rate λ.

The models were implemented in a Bayesian framework using Rstan (35). We used a strongly informative prior for γ, specified as a normal distribution with a mean of 0.5 and a variance of 0.001, and a moderately informative prior for λ, specified as a normal distribution with a mean of 0.1. Parameter posterior distributions were estimated using four chains, each with 3,000 iterations and 900 burn-in iterations. Monte Carlo Markov chain convergence was assessed using Gelman-Rubin R-hat diagnostic with a threshold of 1.1. A threshold of over 400 was used for effective sample size. Means of parameter estimates and 95% credible intervals (2.5% and 97.5% percentiles of posterior distribution) are presented. Model fit is presented in Fig. S4.

## RESULTS

### Participant characteristics and approach to epitope mapping

Our analyses are based on longitudinal serum samples collected from a subsample of 120 children that were selected from the control and the WSH + N intervention group of the WASH Benefits Bangladesh trial (Table S1). Participant characteristics were generally well balanced between intervention and control groups in the substudy. Compared with the intervention group, the control group had more females (57% versus 52%), and mothers had more years of education (6.2 years versus 4.9 years). Overall, household socioeconomic status and WASH characteristics were generally similar between the groups. Measurement age was similar between groups across the three longitudinal visits, with control group children approximately 1 month older on average at visits 1 and 2 (Table 1).

**TABLE 1:** Baseline characteristics of control and intervention groups enrolled in the substudy

Characteristic	Control (*n* = 60)	Nutrition + WSH (*n* = 60)
Demographics		
Sex		
Female	34 (56.7%)	31 (51.7%)
Male	26 (43.3%)	29 (48.3%)
Birth order		
1	25 (41.7%)	14 (23.3%)
2	12 (20.0%)	21 (35.0%)
3+	21 (35.0%)	25 (41.7%)
Missing	2 (3.3%)	0 (0%)
Sampling times		
Visit 1 age (months)^*b*^	4 (1, 6)	3 (1, 6)
Visit 2 age (months)^*b*^	15 (12, 17)	14 (12, 17)
Visit 3 age (months)^*b*^	29 (25, 31)	29 (26, 31)
Household characteristics		
Mothers’ age (years)^*b*^	23.5 (16.0, 31.0)	25.0 (16.0, 39.0)
Mothers’ education (years)^*a*^	6.17 (3.41)	4.85 (3.33)
Fathers’ education (years)^*a*^	5.47 (4.14)	4.37 (3.59)
Number of persons in household^*a*^	4.90 (2.36)	4.52 (1.74)
Number of individuals in compound^*a*^	10.1 (5.45)	11.8 (6.11)
Household is food secure	42 (70.0%)	44 (73.3%)
Wealth index^*a*^^,^^*c*^	0.255 (1.98)	0.427 (1.61)
Household has a television	24 (40.0%)	28 (46.7%)
Household WASH characteristics		
Shallow tubewell is primary water source	40 (66.7%)	50 (83.3%)
Stores drinking water	33 (55.0%)	30 (50.4%)
Primary handwashing station has soap^*c*^	20 (37.7%)	10 (38.1%)
Owns household (unshared) latrine	38 (63.3%)	29 (48.3%)
Latrine has a functional seal^*c*^	17 (32.7%)	14 (27.5%)
Improved floor (wood, concrete)	9 (15.0%)	6 (10.0%)

^*a*^ Mean (SD).

^*b*^ Median (min, max).

^*c*^ Missing data. Percent was calculated from total individuals with data available.

We found that IgG seropositivity had high alignment for PCR positive results (89%), although the sample size is small (*n* = 18). Additionally, a large proportion of IgG-positive samples (82%, *n* = 91) were PCR-negative (Fig. S3). This underclassification is likely due to the short-lived nature of stool-based PCR testing and because IgG incorporates infection over time (36). Additionally, we found that the motifs with highest sensitivity for confirmed *Shigella*/EIEC cases were also detected in the ex-U.S. discovery panel along with 11 additional motifs. These additional motifs likely reflect more mature immune responses from a larger data set of U.S. and ex-U.S. samples compared to *Shigella*/*EIEC* case data, which included only very recent infections. We found that for all 32 reported motifs, 70% or more of WASH samples with the motif were identified as IgG-positive samples using the IWAS classification approach (Table S6).

### Intervention effects on *Shigella*/EIEC IgG

**Fig 2:**
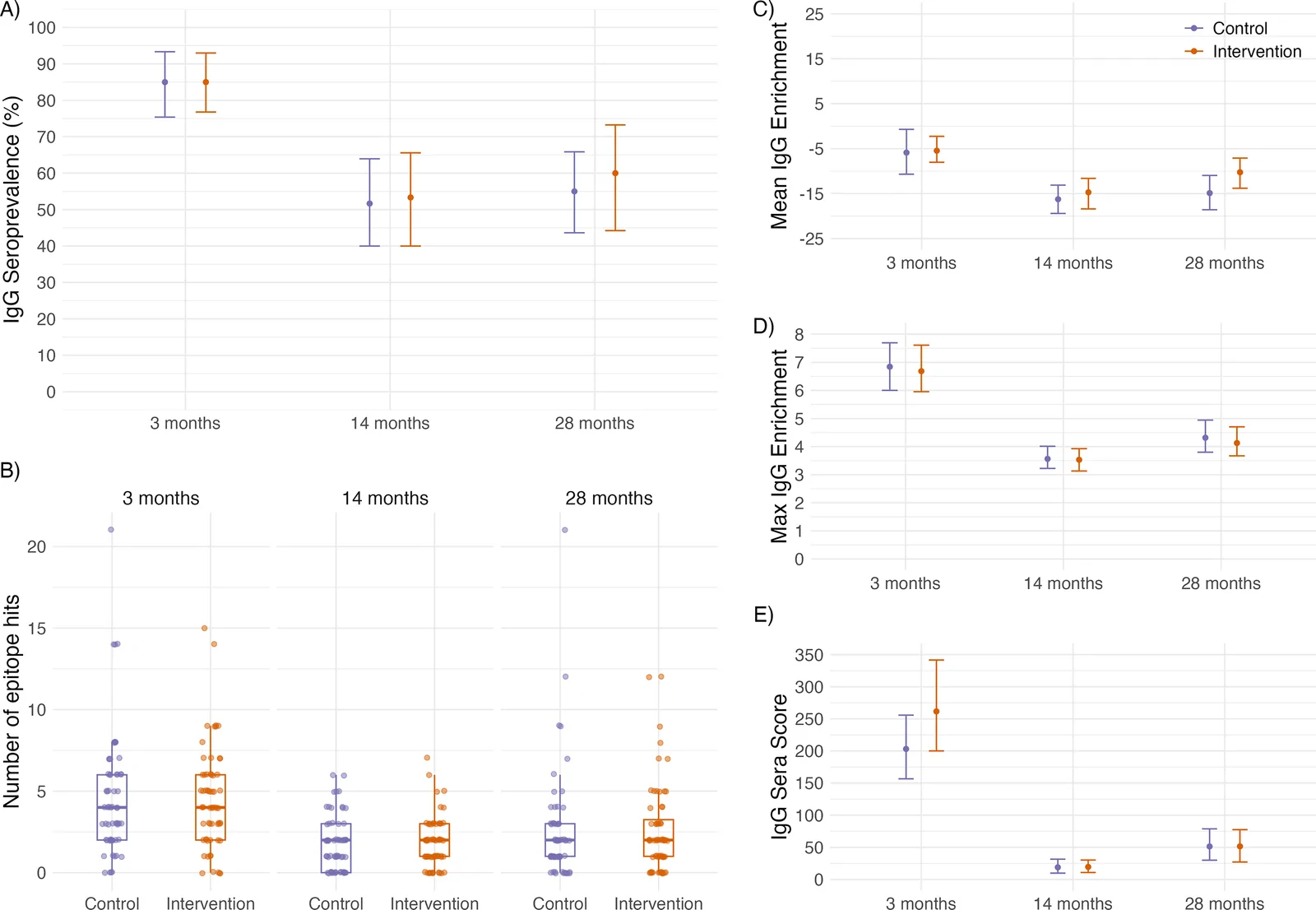
IgG responses to *Shigella*/EIEC Ipa proteins and motifs by intervention group and age. Seroprevalence, epitope counts, and enrichment measures were aggregated across IpaA, IpaB, IpaC, IpaD, and IpaH proteins. Samples were analyzed from three longitudinal visits at median age at 3 (1, 6) months, 14 (12, 17) months, and 28 (25, 31) months (*N* = 60 children per group, 360 total samples). (**A**) IgG seroprevalence by intervention group and age determined as ≥2 epitope hits across Ipa proteins. (**B**) Number of epitope hits across Ipa proteins, by intervention group and age. Box plots summarize the median and interquartile range (IQR), with whiskers representing 1.5 times the IQR. Points represent individual samples (*N* = 60 per group and age). (**C**) Mean IgG enrichment score, calculated as the average IgG enrichment within seroreactive epitope regions of the Ipa proteins. (**D**) Max enrichment score, calculated as the maximum IgG enrichment within seroreactive epitope regions of the Ipa proteins. (**E**) Mean SERA score, calculated as the composite score for epitope motifs identified specific to *Shigella*/EIEC through the IMUNE algorithm. There were no statistically significant differences between intervention groups overall or by age across all IgG summary measures (*P* > 0.05). In panels **A**, **C**, **D**, and **E**, points represent means, and bars represent 95% confidence intervals estimated through bootstrapping, accounting for repeated measures.

We assumed that samples collected at median age 3 months predominantly reflected maternal IgG acquired *in utero* (37, 38). Estimates of intervention effects thus focused on sera collected at ages 14 and 28 months, which we assume measure IgG responses following incident exposures. Seroprevalence in the control group declined from 85% at 3 months to 51% at 14 and 28 months (Fig. 2A), likely reflecting a combination of maternal IgG decay and newly produced IgG as a result of incident exposures, which will be examined in detail below. Age-dependent patterns were nearly identical in the intervention group, and IgG seroprevalence and seroconversion rates estimated across ages 14 and 28 months did not differ between children in intervention and control households (Table S3). Intervention and control groups were also very similar with respect to quantitative measures of immune response, including the number of epitope hits, mean IgG enrichment, maximum IgG enrichment, or IgG SERA score (Fig. 2 and Table S3 and Table S4).

### Age-based patterns of *Shigella*/EIEC IgG epitope repertoire

**Fig 3:**
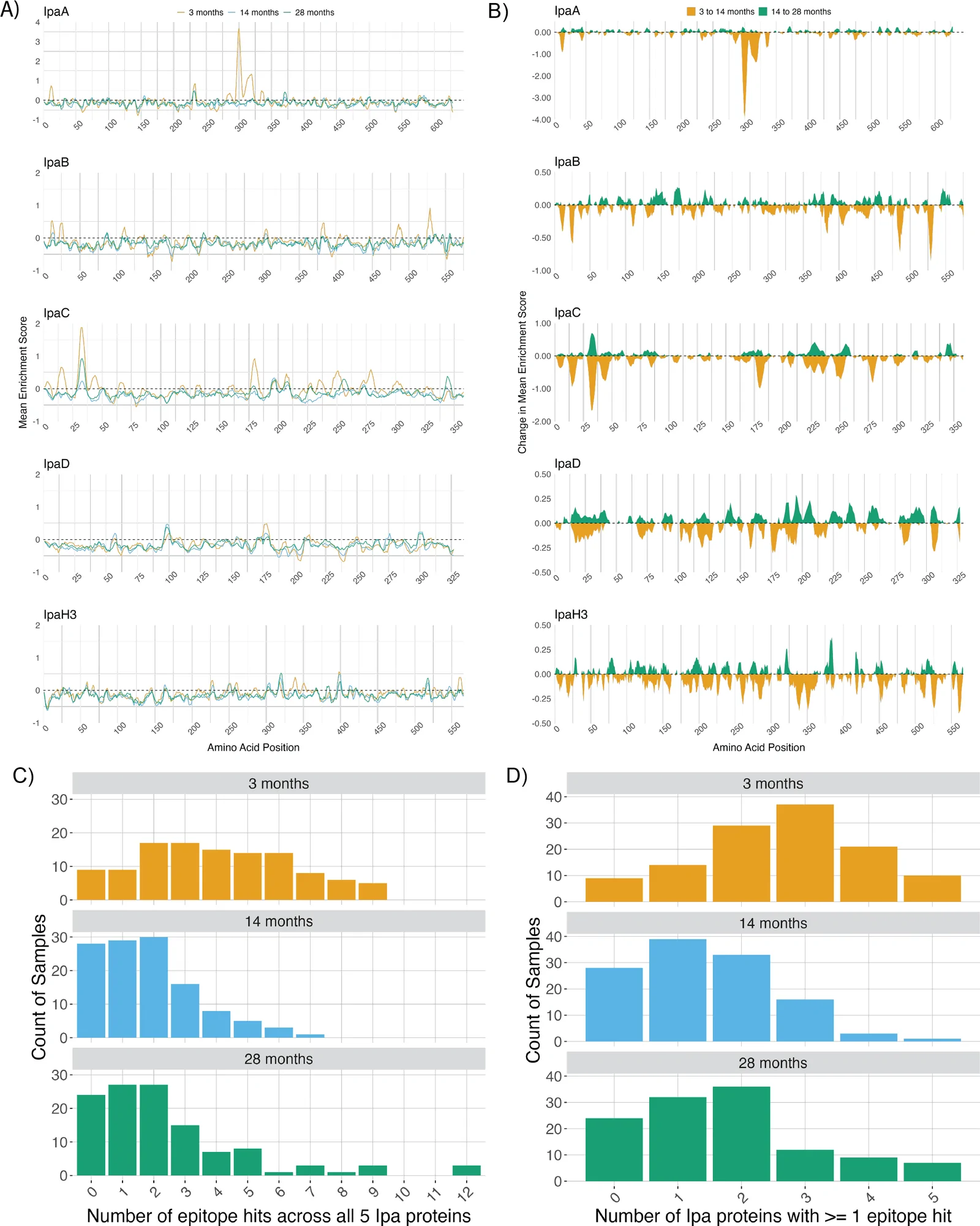
Amino acid enrichment and linear IgG epitope breadth across three longitudinal visits with children’s median age at 3 (1, 6) months, 14 (12, 17) months, and 28 (25, 31) months. (**A**) Measures from the first visit at age 3 months reflect maternal antibodies and show higher enrichment relative to ages 14 (12, 17) months and 28 (25, 31) months. (**B**) The mean change in amino acid enrichment score was calculated by averaging the enrichment score at each amino acid region for all three age groups and then subtracting the calculated mean enrichment score of the preceding visit from the current visit. (**C**) Breadth of Ipa-specific IgG immune response, summarized by number of epitope hits, is higher for 3-month samples. Six 3-month samples (epitope counts: 14 [4], 15 [1], and 21 [1]) and one 28-month sample (epitope count: 21) were excluded to accommodate data visualization. (**D**) Breadth of Ipa-specific IgG responses, defined as the number of Ipa proteins with an epitope hit, is also higher in 3-month samples.

**Fig 4:**
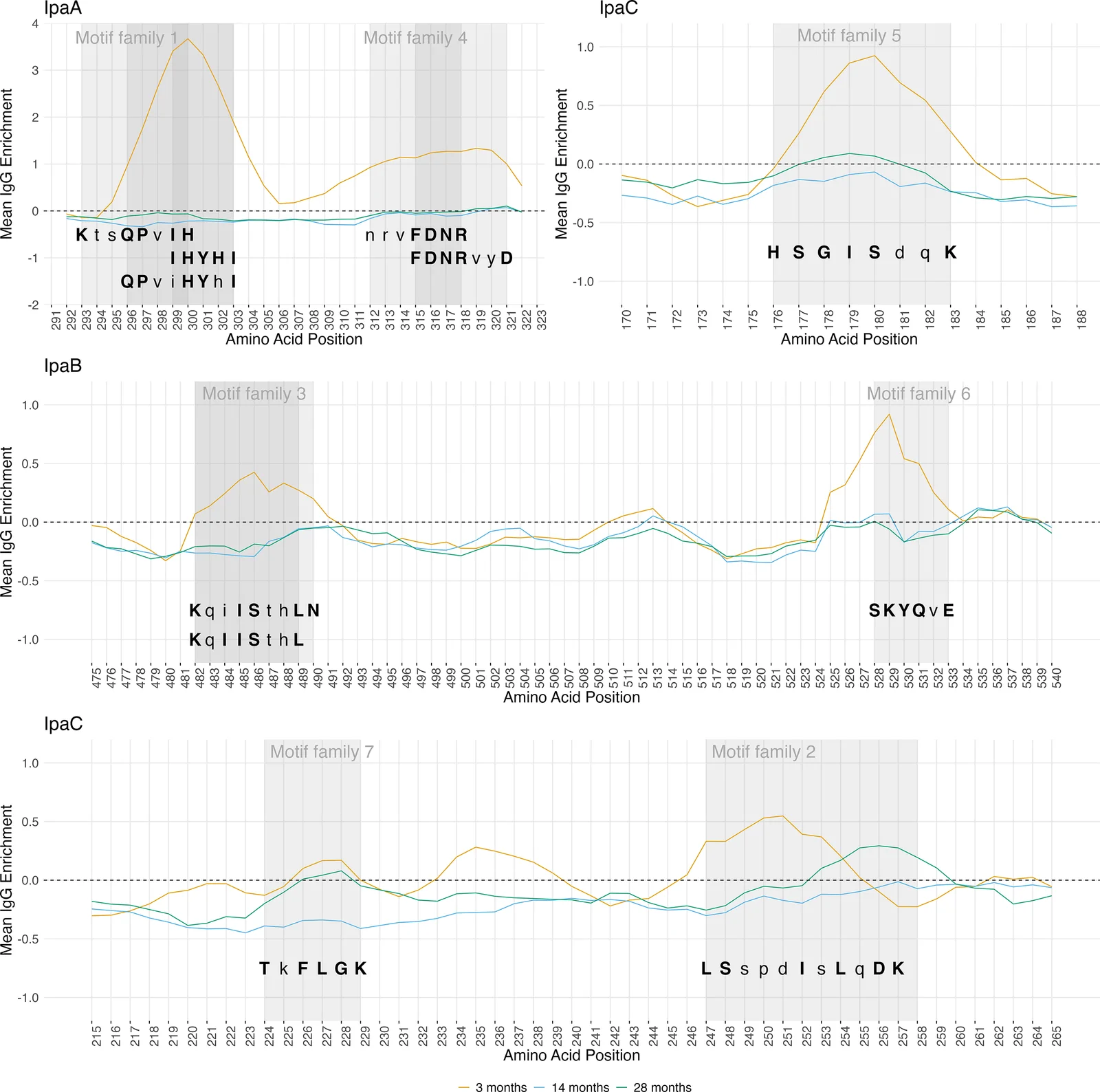
Corresponding amino acid sequences of epitope motif families. Motif sequences were identified using the IMUNE algorithm that compiled *Shigella*/EIEC epitope motifs based on a set of healthy U.S. controls compared to non-U.S. samples. Motif families were defined as identified epitope motifs with overlapping amino acid regions. Motif families were identified in IpaA, IpaB, and IpaC proteins. Gray shading represents the amino acid range of epitopes within each motif family. Capitalized and bolded letters of sequences represent the amino acids of the epitope motifs. Trace lines represent mean IgG enrichment tiling values per amino acid position as determined through the SERA PIWAS tiling method.

Distinct amino acid regions with enrichment for linear epitopes were observed for each of the three ages. Generally, the magnitude of IgG enrichment was higher in 3-month samples, assumed to be the maternally transferred IgG, compared to 14-month and 28-month samples. (Fig. 3A). Notably, IgG enrichment from amino acid positions 290 to 325 in IpaA was four times higher in 3-month samples compared to 14-month samples and did not increase at 28-month samples. The largest epitope motif family 1 corresponded to this amino acid region (Fig. 4). Areas of elevated amino acid enrichment were less distinguished by age for IpaB, IpaD, and IpaH3, though still present. Notably, epitope motifs identified independently through the IMUNE algorithm were present in all Ipa proteins except IpaH3 (Table S5), with motif families, defined as epitope motifs with overlapping amino acid positions, detected in IpaA, IpaB, and IpaC (Fig. 4). The proportion of samples positive for an epitope motif was higher at 3 months compared to 14 months or 28 months for all 32 epitope motifs detected (Table S5).

IgG epitope breadth, defined as the count of epitope hits and the count of unique Ipa proteins with an epitope hit, was higher at 3 months compared to the other time points (Fig. 3C and D). The count of linear epitope hits per sample at 14 months and 28 months was right-skewed, with most samples having only 0, 1, or 2 epitope hits, whereas at 3 months, the distribution of linear epitope hits per child was more uniformly spread over a higher count range. This pattern was also observed for the count of unique Ipa proteins with an epitope hit.

### Inferred *Shigella*/EIEC immunity dynamics and age-based susceptibility

**Fig 5:**
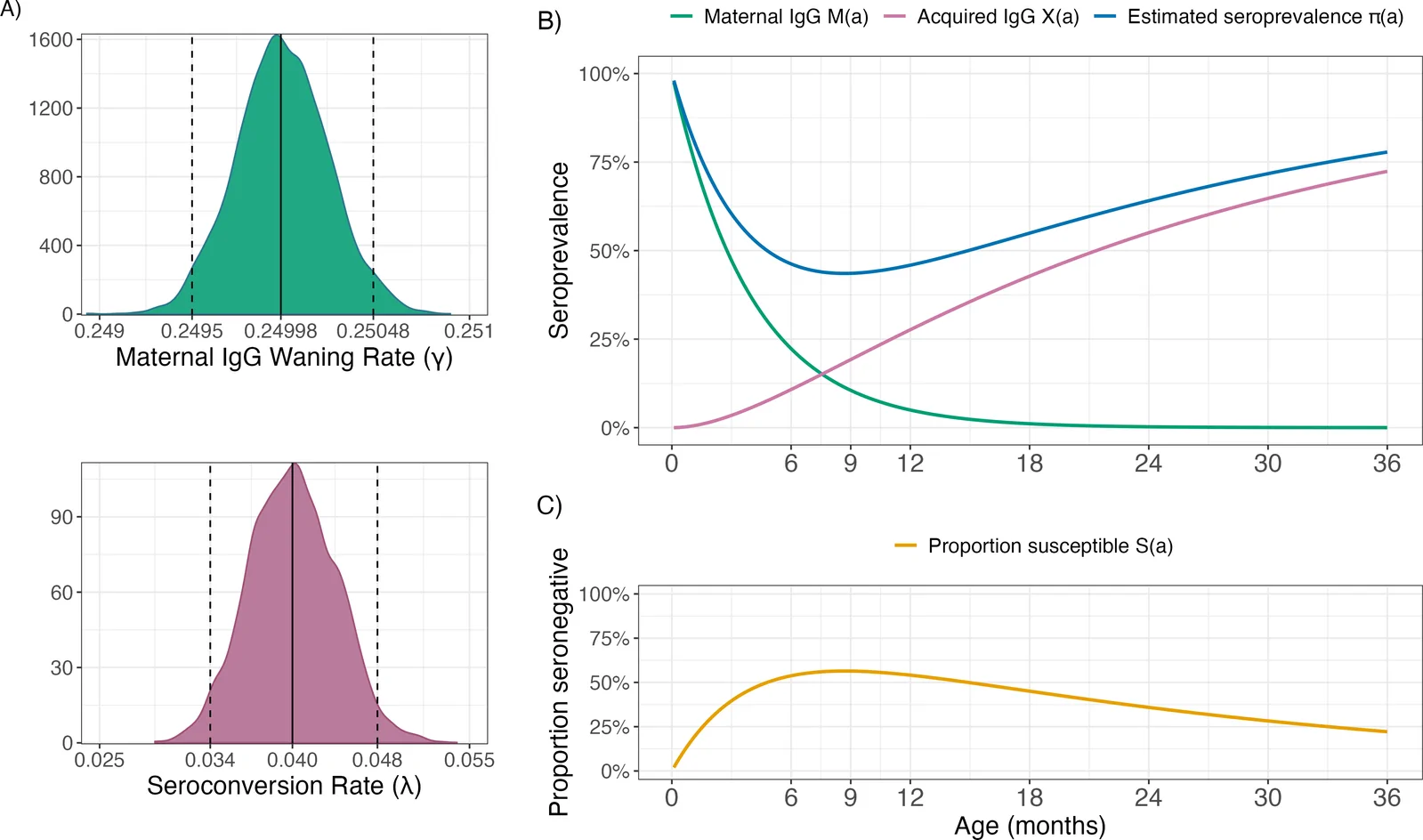
Maternal and child IgG dynamics inferred from age-dependent seroprevalence. A modified catalytic model was used to estimate the rate at which individuals become infected, the seroconversion rate (λ), and the rate at which maternal antibodies wane (γ), under the assumption that all children were born with maternal antibodies. See Materials and Methods for more specifics on model building and mathematical derivations. (**A**) Posterior distributions of model parameters, including the median estimates (vertical line) and 95th credible interval (dashed lines). (**B**) These model parameters were used to estimate population immunity projections, including maternal IgG, acquired IgG, and observed seroprevalence from 1 to 36 months. (**C**) The proportion of the population susceptible to *Shigella*/EIEC, assuming IgG is a correlate of protection, was also estimated from the catalytic model.

We used a modified catalytic model framework to estimate *Shigella*/EIEC exposure rate and maternal antibody waning rate and to model age-specific seroprevalence (Fig. 5). The model estimated IgG seroprevalence steadily declined from 100% seroprevalence to 44% between 6 and 12 months, corresponding with the steady loss of maternal IgG by 12 months. The estimated IgG seroprevalence attributable to maternal IgG approached 0 by 18 months, with a handoff to IgG from natural exposure between 6 and 9 months. Taken together, the estimated proportion of individuals seronegative, or susceptible to infection, peaked at 44% between ages 6 and 9 months.

## DISCUSSION

There was no significant impact of the WSH + N intervention on children’s IgG seroprevalence and seroconversion. Our results suggest that intensive household WSH + N interventions do not influence a child’s immune development to *Shigella*/EIEC Ipa proteins. This is consistent with an absence of intervention effect on *Shigella*/EIEC infection measured by qPCR at age 14 months in the same study population (17). Since IgG integrates information about cumulative *Shigella*/EIEC infections over time, this study provides a comprehensive assessment of intervention effects during the first two years of life. The results suggest that intensive household WASH and nutrition improvements alone may be insufficient to reduce *Shigella*/EIEC transmission and that complementary interventions such as community-level engineered systems that separate human excrement from the living environment, or a vaccine, would be required to meaningfully reduce transmission during the first 2 years of life in high-transmission settings.

Current *Shigella* vaccines in development largely feature the O antigen of the lipopolysaccharide (LPS) as corresponding immunoglobulin G (IgG) antibodies confer protection against shigellosis (17, 18), but more recent studies suggest that the structural Ipa proteins, particularly IpaA, IpaB, IpaC, and IpaD, may be correlates of protection (39–43). Second-generation vaccines have begun to incorporate novel antigens, such as recombinant IpaB, IpaC, and IpaD with LPS, with high efficacy in mice (44–46). Our results suggest that IpaA and IpaC may be important immunogens due to protein regions with high enrichment for linear epitopes. The limited enrichment from IpaB, a common target for vaccine design, relative to IpaA and IpaC, may be because IpaB epitopes are largely conformational and therefore might not have been detected using the SERA PIWAS tiling method, which is trained to detect linear peptides. Despite this, epitope motifs were detected in all Ipa proteins except IpaH3, many of which do not align with epitopes identified through traditional immunoinformatics in previous literature (41). These epitope motif families often corresponded to amino acid regions with high IgG enrichment, suggesting that the SERA and IMUNE pipelines may be detecting novel epitopes not routinely detected through heuristic-driven prediction methods. Validation of these epitopes through experimental methods is needed to confirm this.

In our setting, we estimated that maternal IgG anti-*Shigella*/EIEC Ipa proteins are present at high levels in early infancy and begin to decrease between ages 6 and 9 months. This suggests that childhood vaccination after 9 months may avoid immune blunting from maternal antibodies (47). This vaccine timing implied by IgG decay is similar to transplacental IgG seroprevalence against the lipopolysaccharide (LPS) antigen in a population of Zambian infants, which suggested that the potential vaccination timing would be after 14 and before 52 weeks of age (48). It is also in agreement with a study based in Mali and Ethiopia that determined children in the 12–17 month age group were most vulnerable to *Shigella* exposure using a multiplex immunoassay that included a panel of Ipa and LPS antigens (49). Our findings further support the current consensus that the ideal vaccination window for maximizing protection is before 12 months in high-burden settings. Vaccine crowding, in which multiple vaccines are given in a short amount of time, should be considered as this may diminish immune response to a *Shigella* vaccine (50).

This study had limitations. First, we were unable to characterize non-protein-based epitopes, such as the O-linked antigen of the lipopolysaccharide, due to the limitations of the protein-based immunoinformatic pipeline. Although this is unlikely to have impacted our seroprevalence measure, it is possible that we are underestimating true seropositivity by excluding IgG that is specific for non-Ipa *Shigella*/EIEC targets. Second, we did not investigate other isotypes of antibody responses, such as immunoglobulin A (IgA). IgA is an important mucosal immunoglobulin with similar levels to IgG during infection with *Shigella*/EIEC (51). It is possible that there is additional epitope diversity from IgA that is not included in these results. Nevertheless, a key strength of this study is its randomized intervention assignment and longitudinal design, which allowed us to characterize the epitope repertoire within a community setting beyond laboratory-based animal models or computational prediction-based inferences.

In summary, our findings suggest that WASH interventions alone were insufficient to reduce *Shigella*/EIEC transmission in rural Bangladesh based on IgG immune responses across Ipa proteins. Distinct age-specific antibody patterns over the first 28 months of life provide valuable insights that can inform vaccine development by identifying key motifs and epitopes relevant to the target population and estimating the rate of maternal IgG waning. Taken together, these results highlight the importance of timing for vaccine administration against *Shigella*/EIEC and suggest potential novel epitopes for vaccine design.

## Data Availability

All data needed to evaluate the conclusions in the paper are present in the paper and/or the supplemental materials. All data used to display the figures and the code used for analyses are available at https://osf.io/u92pc under the project DOI 10.17605/OSF.IO/U92PC.

## SUPPLEMENTAL MATERIAL

Legends msphere.00339-26-s0001.docx Captions for Data S1 to S3.

Supplemental material msphere.00339-26-s0002.docx Supplemental tables and figures.

Data S1 msphere.00339-26-s0003.xlsx Data for control panel used in motif discovery.

Data S2 msphere.00339-26-s0004.xlsx Data for motif detection in WASH-B data set.

Data S3 msphere.00339-26-s0005.xlsx Data for *Shigella* case and control panel used in motif discovery.

ASM does not own the copyrights to Supplemental Material that may be linked to, or accessed through, an article. The authors have granted ASM a non-exclusive, world-wide license to publish the Supplemental Material files. Please contact the corresponding author directly for reuse.
