# Supplementary material for: Effects of improved water, sanitation, handwashing, and nutrition on early childhood IgG immune repertoire development against Shigella and enteroinvasive Escherichia coli (EIEC)
Source: mSphere. 2026 Jul 15;11(8):e00339-26. doi: 10.1128/msphere.00339-26 (PMC13508797; doi:10.1128/msphere.00339-26)
Supplement: Supplementary file 1 [file msphere.00339-26-s0001.docx]

Supplemental data 1: Data for control panel used in motif discovery

- Motif sera scores for ExUS and US discovery panel samples

Supplemental data 2: Data for motif detection in WASH-B dataset

- Individual Z-scores for each epitope motif discovered using ExUS/US samples (supplemental data 1)

Supplemental data 3: Data for shigella case and control panel used in motif discovery

- Motif z-scores for discovery panel using confirmed Shigella case and control data
