# Supplementary material for: Effects of improved water, sanitation, handwashing, and nutrition on early childhood IgG immune repertoire development against Shigella and enteroinvasive Escherichia coli (EIEC)
Source: mSphere. 2026 Jul 15;11(8):e00339-26. doi: 10.1128/msphere.00339-26 (PMC13508797; doi:10.1128/msphere.00339-26)
Supplement: Supplementary file 2 [file msphere.00339-26-s0002.docx]

# **Supplemental Tables and Figures**

**Figure S1: Relative composite scores of Ex-US samples to US controls**. Seroprevalence estimates are provided for each group. Sample data originating from the US were used in comparison to samples provided for different infectious diseases prevalent outside of the US (Ex-US). A subset of Ex-US samples was compared with US samples to identify motifs matching known Shigella antigens, resulting in a panel of epitope motifs with a composite summary score shown here.

**Figure S2:** **Sensitivity analysis of amino acid enrichment thresholds**. Different thresholds for the assignment of seroreactive regions, defined by the number of individuals having an epitope hit in that region (at least five individuals or ten individuals), and the number of epitope hits within these assigned seroreactive regions to determine pathogen seropositivity (at least one epitope hit, at least two epitope hits, or at least two different proteins with one epitope hit each) were assessed. Seroprevalence across the three median ages: 3 months, 14 months and 28 months were compared. The final analysis defined seroreactive regions using a threshold of at least five individuals in the study sample having an epitope hit in the amino acid region, and at least two epitope hits in any seroreactive region across all Ipa proteins (navy line).

**Figure S3: Individual child epitope hits between visit 2 (14 months) and visit 3 (28 months)**. Child sets are classified according to PCR status at visit 2 and seropositive status at visit 2 or visit 3. One hundred and twenty total children were classified as PCR+IgG+ (16 children), PCR-IgG+ (75 children), PCR+IgG- (2 children) and PCR-IgG- (27 children). Lines are colored according to whether the child set was seropositive at both time points, seroconverted between the two visits, sero-reverted between the two visits, or remained seronegative, as defined by the epitope hit threshold of two for seropositivity using the SERA method.

**Figure S4: Model fit of IgG dynamics inferred from modified catalytic model**. Black points represent the observed seroprevalence at each age in months from the 120 children in the cohort with 95% confidence interval. Blue points represent the estimated seroprevalence at each age in months, as inferred from the modified catalytic model. The blue line and ribbon represent the simulated model fit as well as the 95% confidence interval over 36 months.

**Table S1**: **Random selection of study participants from the control and intervention groups**. Invention consisted of improved water, sanitation, handwashing conditions as well as improved nutrition (WSH+N).

| **Selection criteria** | **Control (N)** | **Intervention (N)** |
| --- | --- | --- |
| **Substudy population** |  |  |
| Total substudy population | 1788 | |
| **Inclusion criteria** |  |  |
| Children in Control and WSH+N groups | 449 | 451 |
| Blood collected at 3 visits (complete for 2+ visits) | 119 | 173 |
| Children with full sample data available at all three timepoints | 95 | 138 |
| **Exclusion criteria** |  |  |
| Visit 1 measurement between Aug-Nov 2013 | 95 | 93 |
| Age between 28 to 200 days at visit 1 | 83 | 75 |
| Age between 365 to 548 days at visit 2 | 78 | 69 |
| **Final selection*** |  |  |
| Final selection | 60 | 60 |
| **Samples were randomly selected from the sub-pool* | | |

**Table S2**: **Shigella proteins and accession numbers used in panel**

| Protein | Species | Accession number |
| --- | --- | --- |
| IpaA | *Shigella sonnei* | Q3YTQ5 |
| IpaB | *Shigella sonnei* | Q3YTQ2 |
| IpaC | *Shigella sonnei* | Q3YTQ3 |
| IpaD | *Shigella sonnei* | Q3YTQ4 |
| IpaH3 | *Shigella sonnei* | Q3Z2I2 |

**Table S3. Intervention effect: IgG seroprevalence and seroconversion rate.** Seroprevalence was defined as proportion of the population seropositive to *Shigella/*EIEC by having at least two epitope hits across all Ipa proteins (IpaA, IpaB, IpaC, IpaD, IpaH3). Seroconversion estimates based on 60 children in each group measured up to two times at median ages 14 and 28 months. Children who seroconverted by their second visit at median age 14 months were not included in the analysis of incident seroconversions between age 14 months and 28 months.

|  |  |  | **Measure of effect*** | |  |
| --- | --- | --- | --- | --- | --- |
| **Population measure** | **Control**  **(95% CI)** | **Intervention**  **(95% CI)** | **Difference**  **(95% CI)** | **Ratio  (95% CI)** | **p-value** |
| Seroprevalence (%) | 56 (46, 67) | 53 (44, 62) | 3.0 (-10, 17) | 1.1 (0.8, 1.4) | 0.637 |
| Seroconversion rate (per 100 child-years) | 49.4 (39.4, 59.5) | 53.4 (42.4, 64.4) | 0.04 (-0.11, 0.19) | 1.1 (0.8, 1.4) | 0.601 |

*Specific for population measure

**Table S4. Intervention effect: IgG Quantitative measure values.** Mean and max enrichment scores were calculated for amino acids within seroreactive regions, as previously defined in methods.

| **Quantitative measure** | **Control**  **(95% CI)** | **Intervention**  **(95% CI)** | **Difference**  **(95% CI)** | **p-value** |
| --- | --- | --- | --- | --- |
| Mean IgG enrichment score | -0.16 (-0.19, -0.12) | -0.12 (-0.15, -0.10) | 0.03 (-0.0, 0.1) | 0.152 |
| Max IgG enrichment score | 3.94 (3.56, 4.32) | 3.83 (3.46, 4.20) | -0.11 (-0.6, 0.4) | 0.685 |
| IgG sera score | 35.04 (20.37, 49.71) | 35.47 (21.69, 49.25) | 0.43 (-19.7, 20.6) | 0.966 |
|  | | | | |

**Table S5.** **Epitope motifs detected across all Ipa proteins and proportion of samples positive to epitope, by visit**. Motif families are numbered arbitrarily, according to the order they were discovered. Motif families only received a number if more than one epitope motif were detected in overlapping amino acid regions.

| **Shigella Antigen** | **Motif**  **Family** | **Epitope Motif** | **Amino Acid Position** | **3 (1,6)**  **months (%)** | **14 (12,17) months**  **(%)** | **28 (25,31) months**  **(%)** |
| --- | --- | --- | --- | --- | --- | --- |
| IpaA | 1 | [IV]HYHI | 299 - 303 | 58.3 | 0.0 | 2.5 |
|  |  | QPXXHX[NH][VI] | 296 - 303 | 35.8 | 0.0 | 0.0 |
|  |  | QPXXHYXI | 296 - 303 | 29.2 | 0.8 | 2.5 |
|  |  | KXXQPXIH | 293 - 300 | 20.0 | 8.3 | 16.7 |
|  | 4 | FDN[RT]XX[DE] | 315 - 321 | 30.8 | 0.0 | 2.5 |
|  |  | TXXFDNR | 312 - 318 | 3.3 | 0.0 | 2.5 |
|  | - | PX[MF]L[FY]K | 10 - 15 | 33.3 | 4.2 | 12.5 |
|  | - | [NT]XV[FY]DN | NA* | 21.7 | 0.0 | 0.8 |
|  | - | DNX[VI][FY]D | NA* | 19.2 | 0.0 | 0.0 |
|  | - | P[GT]XX[SA]NXK | 286 - 293 | 5.0 | 4.2 | 2.5 |
| IpaB | 3 | KXX[IV]SXXLN | 482 - 490 | 55.0 | 4.2 | 17.5 |
|  |  | KX[VI]ISXXL | 482 - 489 | 46.7 | 2.5 | 12.5 |
|  | 6 | SKYQXE | 528 - 533 | 19.2 | 0.0 | 1.7 |
|  |  | [TS]KYQXE | 528 - 533 | 19.2 | 0.8 | 1.7 |
| IpaC | 2 | LSXXX[LI]XDK | 247 - 258 | 48.3 | 2.5 | 10.0 |
|  |  | LSXXXIX[ILV]XK | NA* | 35.0 | 1.7 | 12.5 |
|  | 5 | [TNS]G[IV]SXXK | 177 - 183 | 24.2 | 2.5 | 9.2 |
|  |  | H[STD]GXSXXK | 176 - 183 | 17.5 | 0.8 | 7.5 |
|  | 7 | TXF[LM]GK | 224 - 229 | 17.5 | 4.2 | 11.7 |
|  |  | TXF[LVMI]GK | 224 - 229 | 17.5 | 4.2 | 12.5 |
|  | - | [AV]GXXLXLN | 201 - 208 | 55.8 | 10.8 | 26.7 |
|  | - | R[YF]XSAXE | 173 - 180 | 40.8 | 2.5 | 9.2 |
|  | - | KXAPXXIS | 231 - 238 | 40.8 | 15.8 | 28.3 |
|  | - | KXXXXGIS | 298 - 304 | 25.8 | 6.7 | 20.8 |
|  | - | Q[TR]NX[TS]XK | 219 - 225 | 23.3 | 6.7 | 6.7 |
|  | - | YXX[IKV]STK | 13 - 19 | 15.8 | 1.7 | 2.5 |
|  | - | PLXV[AG][KR] | 41 - 46 | 15.0 | 5.0 | 2.5 |
|  | - | EIXNTK | 2 - 7 | 13.3 | 1.7 | 3.3 |
|  | - | NAQQ[IV] | 32 - 36 | 24.2 | 2.5 | 14.2 |
|  | - | S[HD]X[GSD]IXXXK | NA* | 21.7 | 0.8 | 7.5 |
|  | - | PD[NG][IV]S | NA* | 1.7 | 0.8 | 0.8 |
| IpaD | - | FXPXX[VCT]NG | 15 - 22 | 22.5 | 2.5 | 1.7 |
| **Due to multiple repeats throughout protein or multiple potential antigens* | | | | | | |

**Table S6.** **Sensitivity of** **epitope motifs detected across all Ipa proteins and proportion of WASH samples positive to motif.** Sensitivity at day 30 post-infection was determined using an independent dataset of known *Shigella/EIEC* cases and controls for a panel of 23 motifs. A panel of 32 preliminary motifs were independently discovered using Ex-US and US samples. Sensitivity data is not available for 11 of the motifs detected exclusively by the Ex-US dataset. Epitope motifs that belong to a family and are depicted in figure 4 of the manuscript are bolded. WASH samples were identified as IgG+ using the IWAS enrichment tiling score.

| **Antigen** | **Motif epitope** | **WASH samples with the motif (%)** | **WASH samples with motif identified as IgG + (%)** | ***Shigella/EIEC***  **sensitivity at day 30 (%)** |
| --- | --- | --- | --- | --- |
| IpaA | **[IV]HYHI** | 20.28 | 91.78 | 86.67 |
| IpaC | **LSXXXIX[ILV]XK** | 16.39 | 81.36 | 86.67 |
| IpaC | SXXX[IV]X[LI]XDK | Not detected | Not detected | 86.67 |
| IpaA | **FDN[RT]XX[DE]** | 11.11 | 100.00 | 80.00 |
| IpaB | **KXX[IV]SXXLN** | 25.56 | 83.70 | 80.00 |
| IpaC | [AV]GXXLXLN | 31.11 | 85.71 | 73.33 |
| IpaA | PX[MF]L[FY]K | 16.67 | 83.33 | 60.00 |
| IpaC | R[YF]XSAXE | 17.50 | 82.54 | 60.00 |
| IpaD | FXPXX[VCT]NG | 8.89 | 84.38 | 60.00 |
| IpaC | KXAPXXIS | 28.33 | 71.57 | 53.33 |
| IpaC | NAQQ[IV] | 13.61 | 83.67 | 46.67 |
| IpaA | [NT]XV[FY]DN | 7.50 | 92.59 | 40.00 |
| IpaC | KXXXXGIS | 17.78 | 71.88 | 40.00 |
| IpaC | **H[STD]GXSXXK** | 8.61 | 90.32 | 33.33 |
| IpaC | EIXNTK | 6.11 | 81.82 | 26.67 |
| IpaC | PD[NG][IV]S | 1.11 | 100.00 | 26.67 |
| IpaA | P[GT]XX[SA]NXK | 3.89 | 85.71 | 20.00 |
| IpaC | Q[TR]NX[TS]XK | 12.22 | 77.27 | 20.00 |
| IpaC | **TXF[LVMI]GK** | 11.39 | 85.37 | 20.00 |
| IpaC | YXX[IKV]STK | 6.67 | 91.67 | 20.00 |
| IpaC | HXPXN[ST]D | Not detected | Not detected | 20.00 |
| IpaB | **[TS]KYQXE** | 7.22 | 88.46 | 13.33 |
| IpaC | PLXV[AG][KR] | 7.50 | 70.37 | 13.33 |
| IpaA | DNX[VI][FY]D | 6.39 | 95.65 | Not detected |
| IpaA | KXXQPXIH | 15.00 | 70.37 | Not detected |
| IpaA | **QPXXHX[NH][VI]** | 11.94 | 97.67 | Not detected |
| IpaA | **QPXXHYXI** | 10.83 | 92.31 | Not detected |
| IpaA | **TXXFDNR** | 1.94 | 100.00 | Not detected |
| IpaB | **KX[VI]ISXXL** | 20.56 | 86.49 | Not detected |
| IpaB | **SKYQXE** | 6.94 | 96.00 | Not detected |
| IpaC | **TXF[LM]GK** | 11.11 | 87.50 | Not detected |
| IpaC | [TNS]G[IV]SXXK | 11.94 | 81.40 | Not detected |
| IpaC | **LSXXX[LI]XDK** | 20.28 | 84.93 | Not detected |
| IpaC | S[HD]X[GSD]IXXXK | 10.00 | 86.11 | Not detected |
